# Alternative Birthing Positions Compared to the Conventional Position in the Second Stage of Labor: A Review

**DOI:** 10.7759/cureus.37943

**Published:** 2023-04-21

**Authors:** Prasiddhi D Satone, Surekha A Tayade

**Affiliations:** 1 Department of Obstetrics and Gynaecology, Jawaharlal Nehru Medical College, Datta Meghe Institute of Medical Sciences, Wardha, IND

**Keywords:** the second stage, maternal birth experience, lithotomy, supine position, alternative birthing positions

## Abstract

The position in which the woman delivers has a lot of impact on the ease of delivery. Women's satisfaction with their birthing experience and the care they receive is significantly impacted by the fact that giving birth is frequently a challenging experience. Birthing positions refer to various postures which can be assumed at the time of delivery by a pregnant woman. Currently, the majority of women give birth either while lying flat on their backs or in a semi-sitting position. Upright positions, which include standing, sitting, or squatting along with side-lying and hands-and-knees, are less common birth positions. Doctors, nurses, and midwives are among the most important healthcare professionals, having a significant influence in deciding which position the woman will give birth in and on the physiological and psychological effects of the experience of a woman in labor. There isn't much research to back up the best position for mothers during the second stage of labor. This review article aims to review and compare the advantages and risks of common birthing positions and know about the knowledge of alternative birthing positions among pregnant women.

## Introduction and background

Birthing positions refer to various postures which can be assumed at the time of delivery by a pregnant woman. Delivering a baby is a lot of hard work and a little uncomfortable too. However, the position in which the patient delivers has a lot of impact on the ease of delivery. Certain positions can make the process of birthing easier during labor. There are a variety of good birthing positions which a patient can be in when it's time to push, and it does not necessarily always be the supine position. Studies have shown that when given the option, women will use a variety of postures, both supine and non-supine [1-3]. In Western nations until the 17th century, giving birth while upright was common [4]. When obstetric tools like delivery forceps were developed in the 18th century, women only gradually began to use supine positions like the lithotomy position [5-7]. Women who have given birth throughout the past few years report frequently using supine positions for labor and birth [8], even though assisted vaginal births are now considerably less common [9]. Alternatives to the supine position have become somewhat more common in the past few decades of the 20th century [10]. After reviewing various studies, the objective of this study is to determine which position is the best and which is the most popular among those in which a pregnant woman can give birth; which position is the best one and which is the most used, as well as various benefits and risk factors associated with alternative birthing positions.

## Review

Methodology

**Figure 1:**
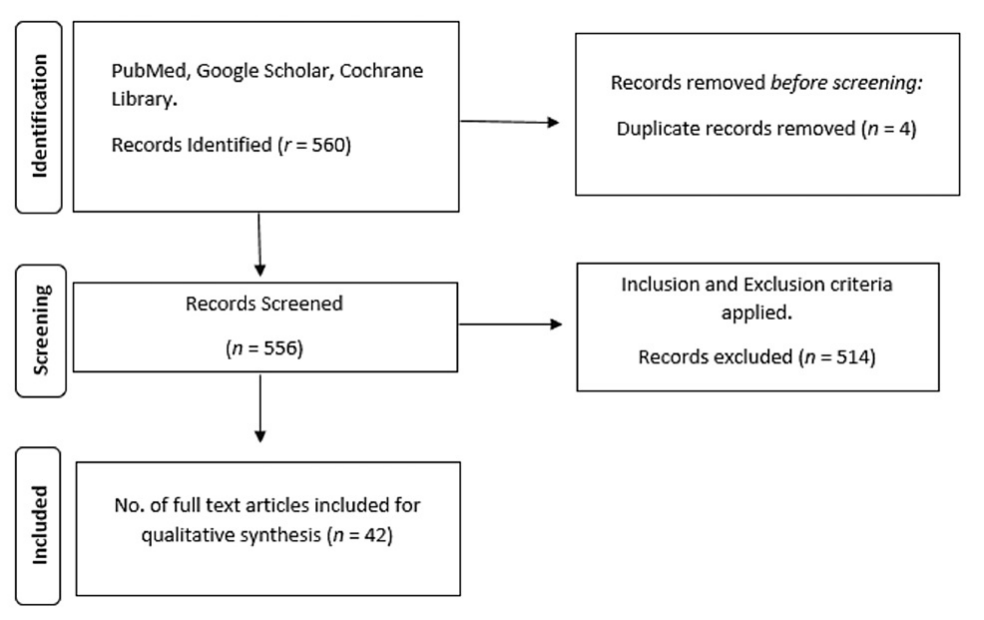
Identification of search strategies for qualitative review synthesis databases and registers on alternative birthing positions in the second stage of labor

We performed searches in electronic databases via PubMed, Google Scholar, and Cochrane Library. The electronic database search was conducted using the following MeSH terms and keyword combinations: ("Alternative Birthing Positions"[Title/Abstract] (("birth s"[All Fields] OR "birthed"[All Fields] OR "birthing"[All Fields] OR "parturition"[MeSH Terms] OR "parturition"[All Fields] OR "birth"[All Fields] OR "births"[All Fields]) AND ("patient positioning"[MeSH Terms] OR ("patient"[All Fields] AND "positioning"[All Fields]) OR "patient positioning"[All Fields])) AND "labor stage, second"[MeSH Terms]). A hand search was also carried out. In addition, we searched the references list for additional studies that might be relevant. The relevant references included in the bibliographies of the studies retrieved from these electronic searches were reviewed. Based on the inclusion and exclusion criteria, 42 studies were finally included in this review for the synthesis of evidence (Figure 1).

Different maternal positions in the second stage of labor

Alternative birthing positions, which include squatting, reclining, sitting, or side-lying, over the conventional posture have definite psychological and physiological advantages. Most women currently give birth either flat on their backs (supine), accounting for 68% of births, or in a semi-sitting position (23%). Upright (standing, sitting, or squatting) (4%), side-lying (3%), and hands-and-knees (1%) are less common birth positions (Table 1) [8].

**Table 1:** Different positions which can be assumed by women during birthing The author created the table.

Different positions
Dorsal supine position
Lateral (Sims) position
Lithotomy position
Semi-recumbent position
Squatting position
Side-lying position
Reclining birth position
Birthing stool position
Birthing bar position
Kneeling position

Research has indicated that compared to a supine position, the duration of the second stage of labor is shorter in an upright position (squatting, sitting, on a birth stool, in a chair, or kneeling). The descent of the fetus is aided by gravity, and the dimensions of the pelvic outlet are also increased in an upright position reducing the chance of labor dystocia [11,12]. The need for episiotomies and assisted deliveries was also seen to be reduced with the upright position [13]. A spontaneous vaginal birth is facilitated by hip flexions, such as that experienced during squatting, which dramatically increase the fetal head angle of advancement via the pelvic axis, cervix, and pelvic floor [14]. When labor and delivery occur in a supine position, the likelihood of cesarean sections was also seen to be increased [13]. Since frequent position changes relieve fatigue, boost comfort, and improve maternal blood circulation, the certainty of the findings is ambiguous.

The preparation of the birth canal is the primary focus of the first stage of labor, which includes the cervix's dilation and effacement as well as the full creation of the lower uterine segment. After the first stage of labor, the second stage of labor begins which includes complete dilatation of the cervix and the expulsion of the fetus through the birth canal [15]. The average duration of the second stage of labor in primigravidae is approximately 50 minutes, and in multiparous, it is approximately 20 minutes but is highly variable [16].

Let us assume a birthing position by a mother for the delivery of a baby vaginally. It is said that women who are moving around tend to have less pain than the ones who are in bed. According to the recommendations of the World Health Organization, an opportunity should be given to pregnant women to choose the type of position she wants to be in during labor [17]. Changing positions during labor and birthing is important for both the mother and the baby and also to make the mother as comfortable as possible (Table 2).

**Table 2:** Different birthing positions The author created the table.

Birthing positions	Description
Dorsal supine position	Lying flat on the back with head and shoulders slightly elevated
Sims position	Lying on the left side with the right hip and knee bent and the left hip and lower extremities straight
Lithotomy position	Lying on the back with knees bent and positioned above the hips and spread apart with the stirrups
Squatting position	Knees and hips bent with the weight of the body on the foot
Kneeling position	The woman kneels, leans forward, and balances herself on her fist or the palms of her hands
Side-lying position	Lying on the side either with legs lifted or supported
Birthing stool position	Sitting up straight on a chair or stool or at an angle of 45 degree
Birthing bar position	Squatting bars that arch over the bed near the foot for support

Supine Position

**Figure 2:**
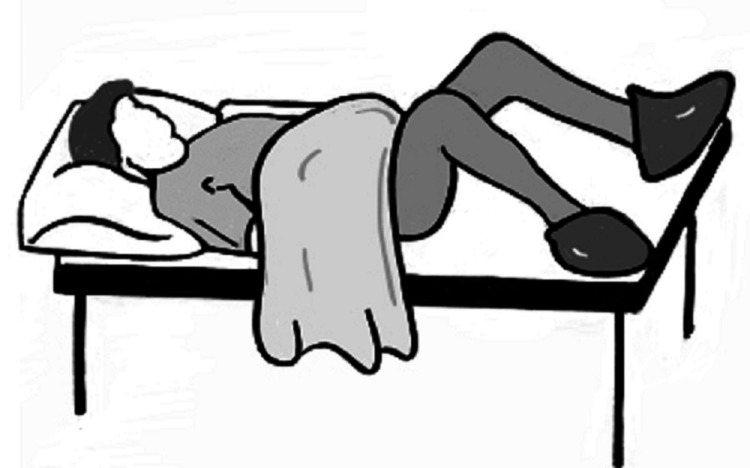
Supine position Author's own creation.

The most common position assumed worldwide by a mother during childbirth is the supine position despite evidence against its use [18]. In this, the woman gives birth on their back and includes dorsal (woman lying flat on her back) (Figure 2), lateral (lying on her side), semi-recumbent, or lithotomy. Due to its prevalence, neither medical professionals nor laboring women anymore consider the supine position to be an intervention. Additionally, the presence of a delivery bed in labor rooms subtly informs women that lying flat is "normal" [17]. These results support research, which found that midwives thought the supine position was the best, most advantageous, and most well-known birthing position [19]. The supine position was associated with a rise in episiotomies [20]. Second-degree tears did tend to decline; however, this was not statistically significant. The rate was greater in the supine position when episiotomies and second-degree tears were coupled to imply perineal injury requiring suturing [10]. The rate of instrumental deliveries was higher in the supine position than in the other positions. Estimated blood loss was lower in the supine position, and postpartum hemorrhage incidence was likewise lower [10]. When a woman is in the supine or lithotomy position during labor, her back mostly supports her weight [15]. This forces the woman to fight gravity and puts the fetus at an unfavorable driving angle concerning the pelvis [21,22]. According to observational studies, lying on one’s back when giving birth may have a negative impact on uterine contractions as the contractions occur frequently but are less effective [21,23], slow down labor, and in, certain cases, limit placental blood flow [23].

Lithotomy Position

The lithotomy position is seen to be used by doctors in many hospitals for both spontaneous as well as assisted vaginal deliveries. The lithotomy position includes lying on the back with knees bent and positioned above the hips and spread apart with the stirrups [5]. The lithotomy position provides the doctor with good access to the mother and the fetus during childbirth. However, this may not be the most comfortable position for the patient. It was the most commonly used birthing position, but recently other positions like squatting, birthing stools, and birthing beds are also being used more often. Studies suggest that a woman delivering in a lithotomy position can experience more pain in the second stage of labor compared to alternative birthing positions [24].

**Figure 3:**
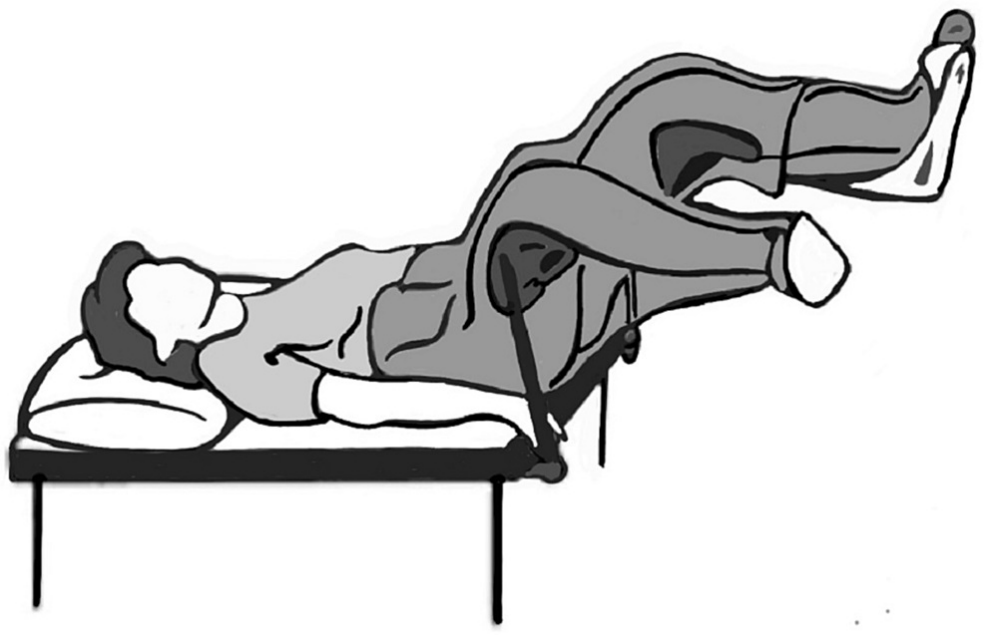
Lithotomy position Author's own creation.

Complications associated with lithotomy position include the increased need for episiotomy and increased chance of forceps delivery or cesarean section [18]. The lithotomy position lowers blood pressure and can increase pain during contractions. It is also associated with an increased risk of perineal injury and more fetal heart rate patterns [13]. While it is convenient for midwives and obstetricians to monitor the progress of labor and perform hands-on interventions as needed while in the lithotomy position, questions continue regarding the hazards of such settings (Figure 3) [25,26].

Lateral Position

Side-lying positions, often known as lateral positions, include the pure side-lying and exaggerated Sims position (semi-prone) [21]. The woman lies on her side in the "pure side-lying posture," either with her hips and knees flexed with a pillow in between the legs or with her legs lifted and supported [21]. The woman assumes the exaggerated Sims position, lying on her side with her lower arm behind (or in front of) her trunk, lower leg extended, and upper hip and knee flexed 90 degrees or more. She then rolls partially toward her front [21]. Additionally, a variation of the lateral position is the Sims position which is also referred to as the left lateral position [27]. When a woman is in the second stage of labor, French midwives prefer lateral positions for both epidural analgesia-treated and non-epidural-treated patients [28].

Squatting Position

Squats are among the popular birthing positions and are also helpful for the induction of labor. In the squatting position, a woman's feet carry the majority of her weight, yet her knees are visibly bent. She may lean or pull on support [5]. The squatting position is frequently viewed as the most natural position, which resembles the way chimpanzees rest and possibly many of us also do [13]. In this position, gravity plays a role during labor as well as delivery. However, maintaining the squatting position for a longer period of time is difficult for pregnant women and, thus, is considered to be one of the major drawbacks [29].

**Figure 4:**
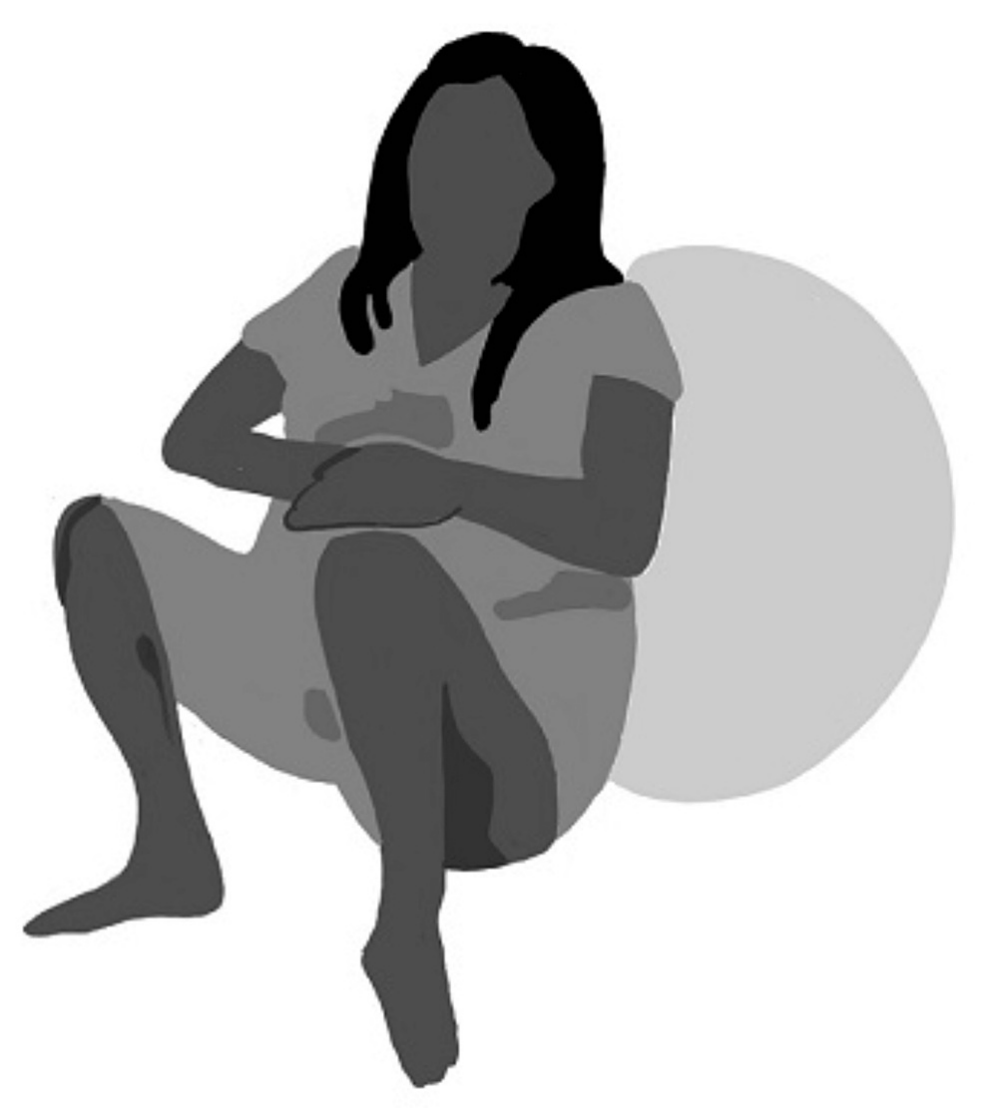
Squatting position Author's own creation.

During the bearing down phase and delivery, it is quite a challenge for the laboring women to maintain a squatting posture, despite research suggesting that it is a natural and, thus, ideal position [29]. This position can put a lot of pressure on the knees and back of the mother and is not easy to maintain. Therefore, the creation of supporting tools may be able to address this issue. According to the findings of a study carried out in Taiwan on the efficacy of ergonomic ankle support aid for squatting position during the second stage of labor, pushing during the second stage of labor puts less stress on the calf muscles of the laboring women when she squats with the help of an ankle supporter [29]. Additionally using a device to aid with squatting decreases the time duration of the second stage of labor, decreases pain, and improves perceived pushing efficiency [29]. Widening the pelvis, the baby has greater space to move in this position. It makes pushing easier by making the body weight press down the uterus (Figure 4).

Birthing Stool

**Figure 5:**
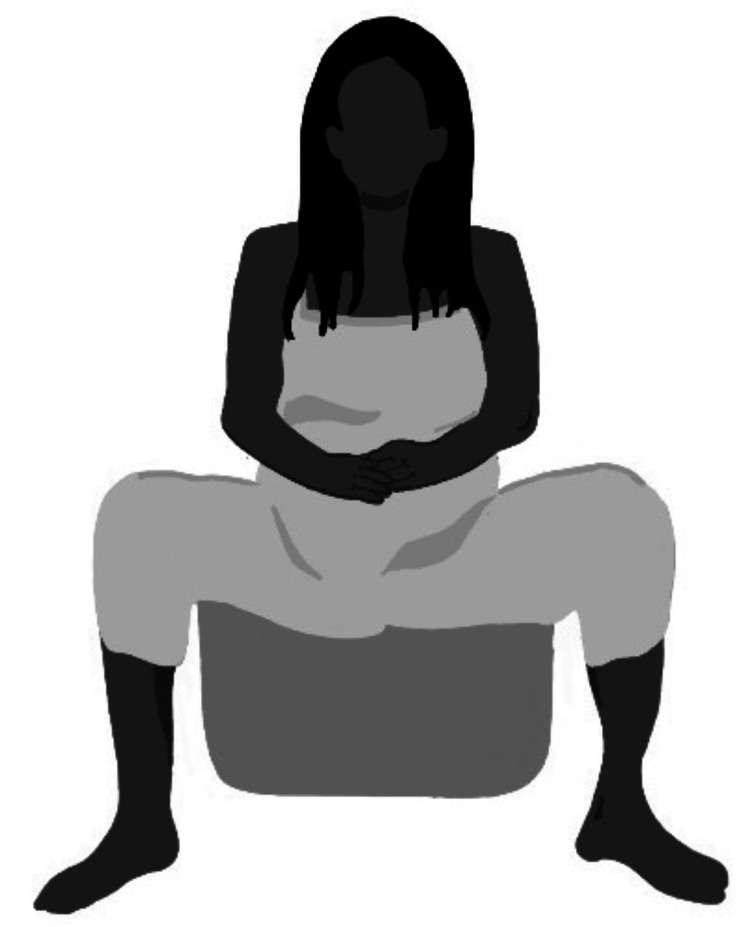
Birthing stool Author's own creation

There are two types of sitting positions: semi-sitting and sitting upright. In the latter, the pregnant woman sits straight up on a bed, chair, or tool, as opposed to the former, where she sits with her trunk at an angle greater than 45 degrees from the bed [21]. Some published research indicates that some Western developed nations appear to favor particular sitting positions more than Asian ones [30,31]. Sitting on a birth seat was the most typical maternal position during the second stage of labor, according to a French study [30]. However, even if they want to, women from various Asian countries have few options for choosing to give birth while sitting down. This is because these cultures frequently practice the position of lying on one's back during birthing [31]. The upright position using the birthing stool helps use gravity to stimulate the baby's downward progress, and the low height of the stool flexes the legs and increases the size of the pelvis. By using a birthing stool, there was a higher risk of blood loss greater than 500 ml (Figure 5).

Birthing Bar

**Figure 6:**
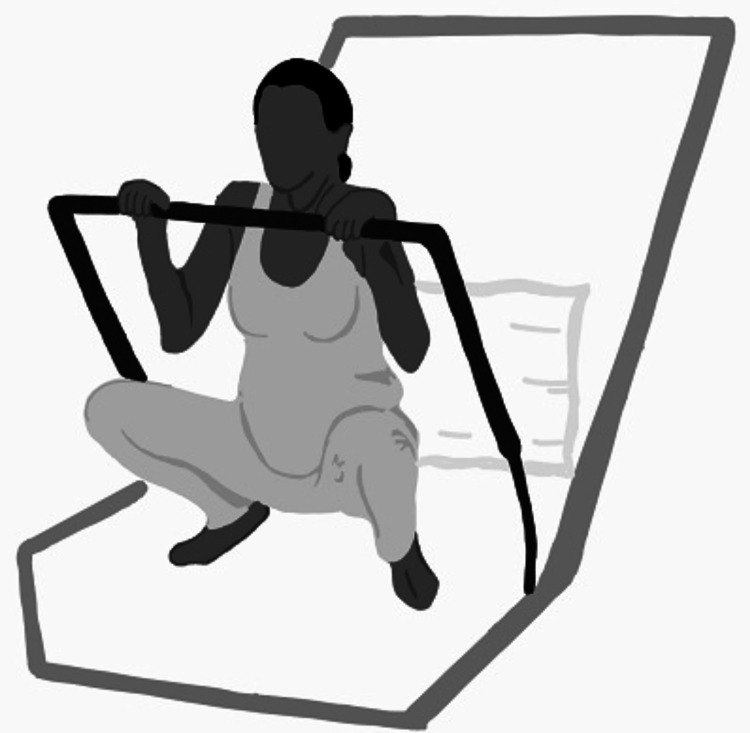
Birthing bar position Author's own creation.

During the pushing phase, squatting bars that arch over the bed near the foot and are secured on each side can be useful. Most labor beds can have a birthing bar attached to them to make it easier to go into a squatting position. The squatting position uses gravity to encourage the baby's downward progress while also expanding the size of the maternal pelvis. When a woman feels a contraction coming, she can lean forward, grab the bar, and pull herself into a squatting position (Figure 6).

Kneeling Position

**Figure 7:**
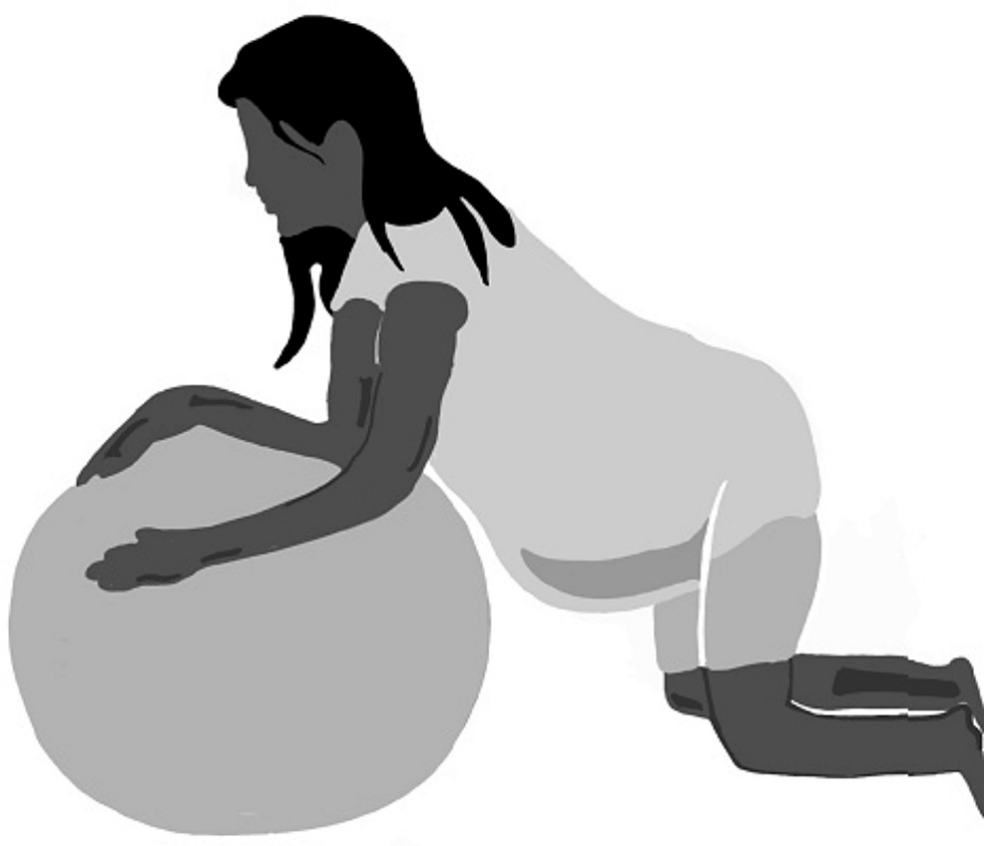
Kneeling position Author's own creation.

Various kneeling positions are possible, including hands-and-knees and upright kneeling [13]. the woman kneels, leans forward, and balances herself on her fist or the palms of her hands in a kneeling position [21]. In comparison to other positions, kneeling positions are less frequent in some Asian countries [31]. If the woman experiences back pain during labor, the kneeling posture may be very helpful because it encourages the baby's movement. Since there is no external pressure on the pelvis, the woman can move more freely while kneeling (Figure 7) [32].

The benefits of giving birth when upright have been well-documented. Aorto-caval compression risk is decreased, the fetus is better aligned, contractions are more effective, and the pelvic outlet is expanded while the woman is in a squatting or kneeling position [10]. Upright positions have been linked to psychological advantages like decreased pain perception, an increased sense of control, more equitable communication with the delivery attendant, and increased partner involvement [33,34]. During delivery, the use of a particular birthing position also varies with parity. The semi-sitting birth position in bed is more frequently used by multiparous women as compared to primiparous women [35]. Regional block analgesia frequently restricts a laboring woman's capacity to move into a different position without help [36].

A meta-analysis of the advantages and risks of various positions during the second stage of labor has been done [37]. The authors concluded that any upright or lateral posture was related to a shorter second stage of labor, less intense pain reported, fewer instrumental deliveries, fewer abnormal fetal heart rate patterns, and fewer episiotomies as compared to supine or lithotomy positions [10,37]. The lateral birthing position also had the highest percentage of intact perineum (66.6% intact, 28.3% lacerations requiring suturing), while squatting was linked to the largest percentage of lacerations (53.2% lacerations requiring suturing, 41.9% intact perineum) as concluded in an Australian retrospective study, which examined the impact of six distinct delivery positions on perineal outcomes, including episiotomy [38]. The lateral recumbent position with its advantage of avoiding compression of the aorta or the inferior vena cava or both is also being used for delivery [13]. Problems for both the mother and the fetus increase when the second stage of labor is prolonged [39,40].

The experience of giving birth is often difficult, and this has a big impact on how satisfied women are with their experience and the care they receive. When a woman is in labor, doctors, nurses, and midwives are among the most important healthcare professionals, playing a significant influence on the physiological and psychological effects of the experience. The mother should be helped to find out which birthing position is the best suitable for her [13]. A study that took place in India shows that around 92% of the nurses working in labor and delivery rooms were aware of the upright birthing positions, and most of them, about 83%, believed that women should be given the choice of whether to deliver in an upright or supine position. However, all of the nurses (100%) said that the most commonly used birthing position was the lithotomy position because of the ease and convenience of the doctors and health care providers [17]. The understanding of several positions, including standing, squatting, lateral, sitting, and hands-and-knees, was implicitly lacking. About 50% of the nurses were familiar with the squatting position, 37% sitting, 23% lateral, 23% hands-and-knees, and 13% standing among the different alternative categories of birthing positions [17]. Some evidence-based guidelines encourage and support women to move and take any position they feel most comfortable with throughout labor and delivery, as opposed to supine or semi-supine positions [41-43].

## Conclusions

There is strong evidence that second-stage labor should not be performed while the mother is in the supine position. Supine positions are linked to greater fetal heart rate abnormalities and fewer spontaneous vaginal deliveries than upright or side-lying positions. When the second stage is prolonged or an expedited birth is necessary, squatting or sitting may be advantageous, while side-lying or hands-knee positions may help prevent lacerations. Despite the proven advantages of giving birth in an upright position, most women deliver vaginally lying on their back in lithotomy, semi-sitting, or semi-recumbent position. In addition, only a small portion of women use alternative birthing positions. As it is more convenient for health care providers to deliver in supine or semi-sitting positions, it is thought that they are the ones who encourage mothers to give birth in these positions.
